# Low-Cost Localized Surface Plasmon Resonance Biosensing Platform with a Response Enhancement for Protein Detection

**DOI:** 10.3390/nano9071019

**Published:** 2019-07-16

**Authors:** Yun Liu, Ning Zhang, Ping Li, Li Yu, Shimeng Chen, Yang Zhang, Zhenguo Jing, Wei Peng

**Affiliations:** 1 School of Physics, Dalian University of Technology, Dalian 116024, China; 2 School of Optoelectronic Engineering and Instrumentation Science, Dalian 116024, China

**Keywords:** localized surface plasmon resonance, biosensing, gold nanoparticle, capillary

## Abstract

There are many potential applications for biosensors that can provide real-time analysis, such as environmental monitoring and disease prevention. In this study, we investigated a simple strategy for real-time protein detection, which had the advantages of affordability, fast response, portability, and ease of use. A robust quantification of protein interaction was achieved by combining capillary localized surface plasmon resonance (LSPR) sensors and complementary metal–oxide–semiconductor (CMOS) image sensors. Gold nanoparticles were modified on the inner wall of the capillary, which was used as a microfluidic channel and sensing surface. We functionalized one of the LSPR sensors using ligand bound to gold nanoparticle. Our proposed biosensing platform could be easily multiplexed to achieve high throughput screening of biomolecular interactions, and it has the potential for use in disposable sensors. Moreover, the sensing signal was enhanced by the extinction effect of gold nanoparticles. The experimental results showed that our device could achieve qualitative identification and quantitative measurement of transferrin and immunoglobulin G (IgG). As a field-portable and low-cost optical platform, the proposed LSPR biosensing device is broadly applicable to various protein binding tests via a similar self-assembly of organic ultrathin films.

## 1. Introduction

At present, the plasmonic biosensor has become one of the most anticipated and reliable characterization approaches for a range of fields, such as analytical, medical, environmental, edible, and pharmaceutical sciences [1,2,3,4,5]. Over the years, there has been continuously increasing interest in ubiquitous, portable, low-cost, and rapid detection devices for optical biosensing [6,7,8]. However, the current plasmonic biosensors have a series of unfavorable factors, including high cost, bulkiness, and complicated processing [9,10,11,12]. To make these plasmonic sensors more suitable for field or in-situ biosensing, there is increased focus on miniaturized and portable plasmonic sensors [13,14]. For example, after being investigated, fiber-optic probe-based surface plasmon resonance (SPR)sensors have attracted significant interest citing the advantages of sensing element miniaturization, reusability, small sample volume, simplified optical design, and the capability for remote sensing [15,16]. Moreover, fiber-optic LSPR sensors have been developed to simplify the fabrication process and to improve sensing performance [17,18]. Gold nanoparticles (GNPs) are used to replace the gold film as the sensitive layer, and they are modified on the fiber surface through layer-by-layer self-assembly [19]. The manufacture of fiber-optic (FO) LSPR sensors is much easier than manufacturing FO SPR sensors because it does not require gold film deposition, which often relies on expensive coating machines, such as evaporation equipment or a magnetron sputtering machine [20,21]. For biosensing applications, the LSPR sensor has different mechanisms to the SPR sensors, and it is more sensitive to the small variation of refractive indices (RI) [22]. With the exception of the traditional SPR sensor and nanoparticle LSPR sensor, the plasmonic nanostructure biosensor with a high figure of merit and enhanced sensitivity has also been widely reported [23,24]. However, its nanofabrication procedure is expensive and time-consuming, thereby hindering practical application and batch manufacturing. Although plasmonic sensors have reduced the volume and cost, as well as simplified the operation process, a further reduction in cost, size, and ease of use is still required for their ubiquitous application. 

Herein, we developed a compact and economical plasmonic sensing platform where the capillary LSPR sensor was integrated with CMOS image sensors for the qualitative identification and quantitative detection of proteins. The LSPR sensor was constructed through modification of the inner wall of the capillary with self-assembled GNPs. The sensing device can be easily multiplexed to enable high-throughput screening of biomolecular interactions, and it has the potential for use in disposable sensors. Our device was used to detect transferrin and immunoglobulin G (IgG), which are important proteins in the human body [25,26]. Transferrin is responsible for transporting iron from the sites of absorption to tissues throughout the body. Its level is used as an indicator of the liver function test for physical examination. IgG is the most common type of antibody found in the blood circulation. The measurement of IgG can be a diagnostic tool for certain conditions, such as autoimmune hepatitis. Many methods have been proposed for diagnostic determinations of the proteins in the human body, such as immunochemical nephelometry, capillary zone electrophoresis (CZE), piezoelectric immunoassay, immunological turbidimetric assay (ITA), radioimmunoassay (RIA), as well as the electrochemical immunoassay [27,28,29,30,31]. Moreover, these methods are costly and limited to research institutes or medical facilities in places such as universities, hospitals, and research centers. Therefore, this study aimed to develop an affordable, portable, and easy-to-use device for the detection of proteins, which has potential to be adapted to daily life and field application.

## 2. Principles and Structure

### 2.1. Characteristics of the Gold Nanoparticle

**Figure 1:**
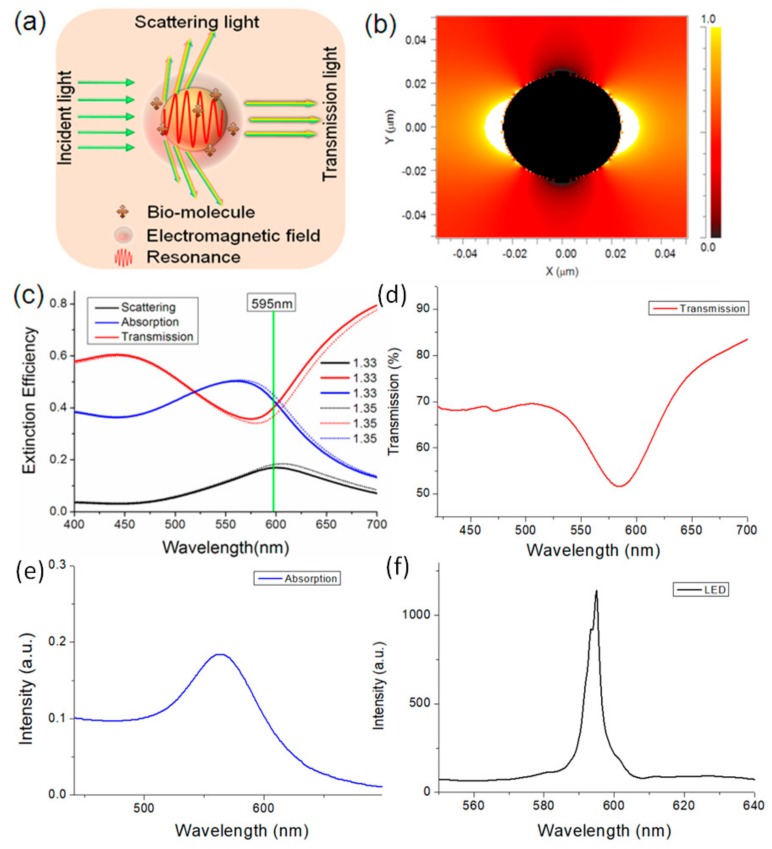
(**a**) Extinction of the gold nanoparticles (GNPs); (**b**) Electromagnetic field distribution on the AuNP surface with a surrounding RI of 1.333; (**c**) Extinction spectra of the GNPs. (**d**,**e**) Transmission and absorption spectra of the gold nanoparticle. (**f**) Emission spectrum of the LED.

Both scattering and absorption cause the extinction of light for the gold nanoparticles (Figure 1a). The extinction efficiency of the GNPs is highly dependent on the dielectric properties of the surrounding environment. To investigate the extinction of the GNPs, the finite-difference time-domain (FDTD) method was used to calculate the electric field around a GNP with a surrounding refractive index (RI) of 1.333. The diameter of the GNP was 40 nm and the wavelength of light was 595 nm. The cross section of the GNPs for a range of irradiances is shown in Figure 1b. We find that the area with high intensity is the surface of the GNP. It means that power absorption is caused by localized surface plasmons (LSP) and the LSPR effect near the surface of the GNP. Meanwhile, some weak power is distributed around the nanosphere in all directions, which is caused by scattering of the GNPs. We simulated the absorption efficiency function and scattering efficiency function of the GNPs ranging from 450 nm to 750 nm. As shown in Figure 1c, both the absorption efficiency and the scattering efficiency have peaks near 600 nm, whilst the absorption efficiency is much higher than the scattering efficiency. We also simulated the theoretical RI response of the GNPs by changing the RI parameters from 1.33 to 1.35. After increasing the RI, both the absorption efficiency and the scattering efficiency increased. It meant that a higher surrounding RI lead to stronger power absorption and scattering, which influenced the intensity of the transmission and scattered light of the GNPs. Figure 1d,e show the measured transmission and absorption spectra of the gold nanoparticle, where the wavelengths of the transmission dip and the absorption peak were in the range of 570–600 nm. Figure 1f shows that the center wavelength of the light-emitting diode (LED) is about 595 nm, which is in the range of the transmission dip and the absorption peak. As a result, it was possible to estimate the surrounding RI by detecting the transmission power and the brightness of the GNPs under illumination.

### 2.2. Functionalization of the LSPR Sensor

**Figure 2:**
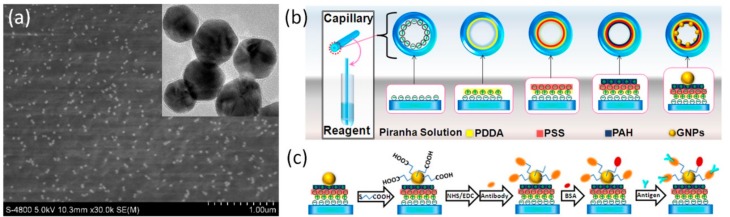
(**a**) Scanning electron microscope and transmission electron microscope of the gold nanoparticles. (**b**) Fabrication process of the capillary-based localized surface plasmon resonance (LSPR) sensors; (**c**) surface modification of the GNPs and binding of antibody/antigen.

We fabricated plasmonic sensors using GNPs and capillaries (TSP, 250/350 Molex), which were used as surface plasmon resonance sensors in our previous works [32,33]. The length of the capillaries used in this work was 1.5 cm. GNPs assembled film as a sensing layer were built on the polyelectrolyte multilayer modified inner surface of the capillary (Figure 2a). Figure 2b shows the scheme illustrating the steps to modify the GNPs to the sensing surface. First, we removed the coating of the capillaries and then cleaned them with acetone in an ultrasonic bath for 10 min. Then, the capillaries were treated with Piranha solution [a mixture of hydrogen peroxide (30%) and sulfuric acid (70%) for 90 min], followed by flushing with deionized water. Polyelectrolyte trilayer was selected as a linker for self-assembly of the GNPs by the electrostatic interaction. After assembly of the first-layer diallyldimethylammonium chloride (PDDA), the inner surface was positively charged. Then, sodium-p-styrenesulfonate (PSS) and allylamine hydrochloride (PAH) were sequentially assembled on the capillary surface. With a positively-charged PAH layer on top of the inner surface, the capillaries were filled with gold colloid for 120 min to form self-assembled monolayers of GNPs. All of the chemical agents used to treat the inner surface of the capillary were introduced into capillaries through the capillarity phenomenon.

To functionalize the capillary LSPR sensors, we immobilized a biofilm of anti-transferrin on the surface of the GNPs. The functionalized process was performed as depicted in Figure 2c. To introduce carboxyl groups on the surface of the GNPs, 11-mercaptoundecanic acid (MUA) in 1 mM ethanol solution was injected into the capillary LSPR sensor to let the nanoparticle react with the MUA for 12 h. After being washed with distilled water, the modified capillary LSPR sensor was soaked in an aqueous solution containing N-hydroxysuccinimide (NHS, 0.5 M) and 1-ethyl-3-[3-dimethylamino-propyl] carbodiimide hydrochloride (EDC, 0.55 M) at 4 °C for 30 min. Then, the capillary LSPR sensor was washed with distilled water and dried with nitrogen. Subsequently, the treated capillary LSPR sensor was dipped in an anti-transferrin/protein A solution of 0.1 mg/mL for 30 min. Then, the capillary LSPR sensor was rinsed with 0.01 M phosphate-buffered saline buffer (PBS, pH 7.4) and blocked using bovine serum albumin (BSA, 0.1 mg/mL) at ambient temperature for 15 min.

### 2.3. Principle of the Biosensing Device 

**Figure 3:**
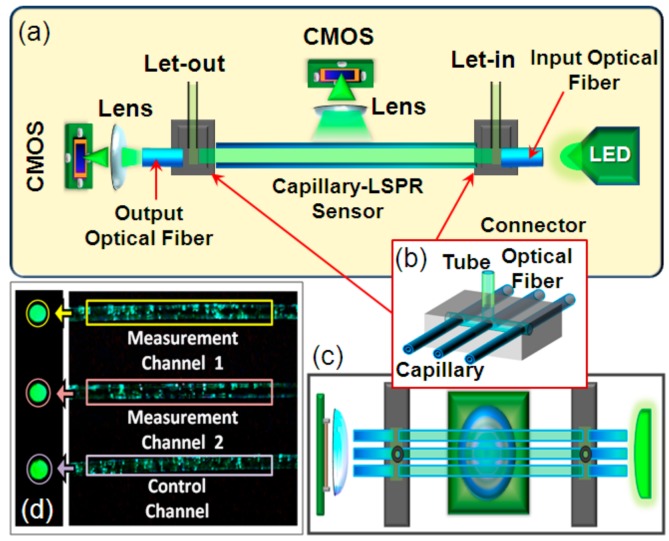
(**a**) Schematic diagram of the plasmonic sensing device based on capillary LSPR sensors; (**b**) Structure of the connectors; (**c**) Vertical view of the sensing device; (**d**) Image detected by CMOS.

The structure of the plasmonic sensing device is schematically illustrated in Figure 3a,c. Two connectors were used to connect the optical fibers and the functionalized capillary-LSPR sensors as shown in Figure 3b. The optical fiber used in this experiment was plastic cladding multimode fiber, with a core diameter of 400 μm and a numerical aperture of 0.37. The ends of the optical fibers were placed as closely as possible to the LED. The emission wavelength of the LED light source was selected to be 595 nm, which fell into the strong absorption and scattering bands of the capillary-LSPR sensor. All end faces of these capillaries and optical fibers were polished with emery papers to be smooth. Two CMOS image sensors connected to a laptop (640 × 480 pixel) were used to monitor the transmission light from the output optical fibers and scattered light from the sidewall of the capillaries, respectively. As shown in Figure 3d, the images of the optical fibers facets and capillary walls were displayed as light spots and bright areas on the screen. We selected the sidewalls and fiber end-faces as regions of interest (ROI). The total intensity of each ROI was used to calculate the response of the capillary LSPR sensor. For ROI selection, we designed an image processing program based on Labview, data acquisition, data storage, and data processing in real time. The images were captured and processed in real time whilst the program was running. The brightness information was extracted from the image data through a conversion of the colored image to grayscale images. The intensity of each ROI was calculated as the mean of grayscale value for all pixels in the ROI. 

## 3. Experiments and Results

### 3.1. Self-Reference Function Test

**Figure 4:**
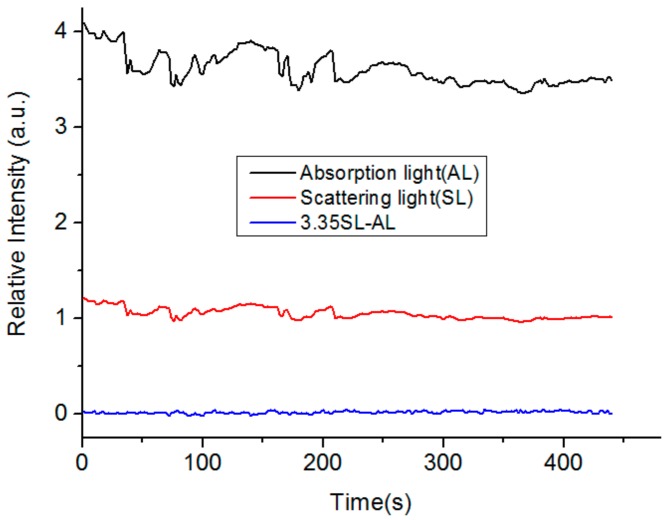
Intensity fluctuation of the plasmonic device based on capillary LSPR sensors.

Since we used the CMOS image sensors to detect the scattering and transmission light of the capillary, the intensity variation of the light source influenced the results. Thus, we tested the responses of scattered light and transmission light on the LED intensity fluctuation. We changed the LED intensity and recorded the responses of scattered light and transmission light in real time. We monitored the scattered light and transmission light of the capillary sidewalls and fiber end-faces using two CMOS image sensors at the same time. As the intensity of the LED fluctuated, the scattered light from the sidewalls of the capillaries and transmission light of the fiber end-faces were flickering. Figure 4 shows the experimental results where the two variables have a simultaneous change trend. Moreover, we found that the response of scattered light was 3.35 times more than that of the transmission light. Therefore, the intensity difference between the scattered light of the sidewalls and the transmission light of the fiber end-faces was used as the measured signal, which was calculated as ΔI = 3.35I_sca_ − I_tra_. Where, I_sca_ and I_tra_ are the intensities of scattered light and transmission light, and ΔI is the difference between I_sca_ and I_tra_. The intensity fluctuation of the light source affects both the scattered light and the transmitted light of the gold nanoparticles in the same way. Thus, if you only consider the intensity fluctuation of the light source, in theory, the rate of light intensity change (ratio R between the changed light intensity ∆I and the initial light intensity $I_{initial}$) for scattering light ($R_{sca}=∆I_{sca}/I_{initial-sca}$) and transmitted light ($R_{tra}=∆I_{tra}/I_{initial-tra}$) should be identical. Assuming $kI_{initial-sca}=I_{initial-tra}$ (where k is a constant), we can get $k(I_{initial-sca}+∆I_{sca})=I_{initial-tra}+∆I_{tra}$, i.e., $kI_{sca}=I_{tra}$. However, the RI fluctuation has different influences on the scattered light and transmitted light. It meant that we could eliminate the fluctuation of the light source and maintain the RI response by comparing the light intensities of the scattered light and transmitted light of the nanoparticles. In this experiment, the k was 3.35 and we used the $∆I=3.35I_{sca}-I_{tra}$ as the response signal. Thereafter, we applied the processing algorithm to the results shown in Figure 4. As a result, the influence of intensity variations caused by LED intensity fluctuations could be effectively weakened from the response of I_sca_ and I_tra_.

**Figure 5:**
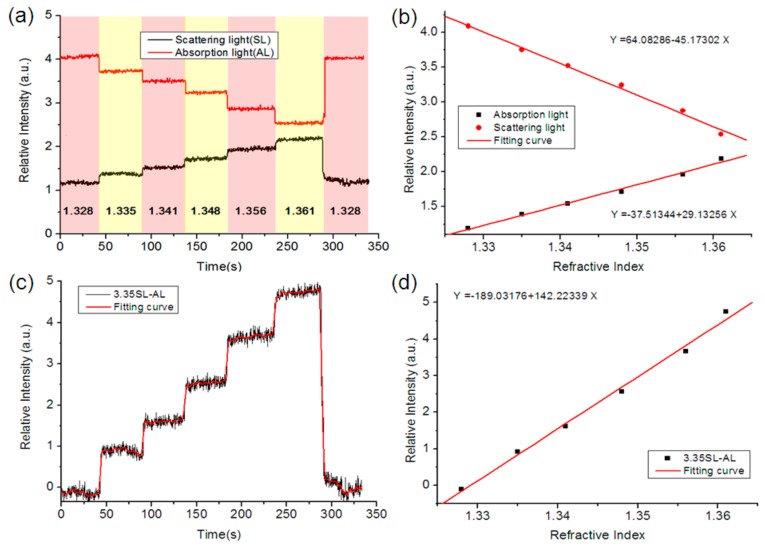
(**a**) Real-time region of interest (RI) response; (**b**) Relationship between the RI and the intensity; (**c**) Real-time RI response based on the processing algorithm; (**d**) Relationship between the RI and the intensity.

Then, we also tested the RI responses of the scattered light and transmission light with sodium chloride solutions of 1.328, 1.335, 1.353, 1.361, and 1.328 RI values. In the experiment, we injected reagents into the capillaries through the let-in connector. Figure 5a describes the real-time change of relative intensity of the device and its RI response. By introducing a sodium chloride solution with a higher RI, we observed an increase of relative intensity in the scattered light. In contrast, for the transmission light of fiber end-faces, an obvious decline of relative intensity was recorded at the same time. We selected the mean of relative intensity in a stable state as the measurement signal for each solution. By fitting the data, the relationship between the RI and the relative intensity was found to be approximating linear. We also applied the algorithm (ΔI = 3.35I_sca_ − I_tra_) to the RI responses. By calculating the intensity difference between the scattered light of the sidewalls and the transmission light of the fiber end-faces, the RI response of relative intensity was magnified as shown in Figure 5c. 

The above experimental results showed that the response tendencies of scattered light and transmission light were opposite for RI change, whilst they were identical for intensity variation of the LED. Thus, it was possible to enhance the RI response and weaken the influence from intensity fluctuation using the ΔI as the measurement signal. However, except for the intensity fluctuation from the light source, the bulk RI variation also influenced the biosensing response. Therefore, one of the capillaries modified with GNPs was used as a control channel to detect the bulk RI, whilst the other two capillaries modified with GNPs and a biomolecule probe were used as the measurement channels. By using the relationship between intensity and the RI as shown in Figure 5d, we converted the intensity value of each capillary sensor to the RI value as the responses. Then, we obtained the measurement signal without the bulk RI by subtracting the response of the control channel from the response of the measurement channel, which could be expressed as ΔR = R_mea_ − R_con_. Where, R_mea_ and R_con_ are the responses of the measurement channel and control channel, and ΔR is the difference between R_mea_ and R_con_. 

### 3.2. Real-Time Biosensing

We used the proposed device to detect proteins and monitor the binding process between an antibody and antigen (transferrin protein and anti-transferrin/IgG and Protein A). Considering that the optical properties of gold nanoparticles were not very sensitive to temperature variation, this biosensing experiment was completed at room temperature of 25 °C. As samples, transferrin/IgG was serially diluted in PBS buffer from 0.01 mg/mL to 0.15 mg/mL. First, the PBS buffer was injected into the capillaries as reference, and the response of each channel was constantly monitored and recorded to construct the baseline. We continuously injected the buffer into the capillary LSPR sensor to establish a baseline signal. After the baseline was stable, we injected the transferrin samples into the capillary LSPR sensor. As the antibody (transferrin/IgG) was binding to the antigen (anti-transferrin/protein A) on the GNPs surface and leading to an RI increase, we found that the ROI responses of the sidewalls of the capillaries increased whilst the ROI responses of the fiber end-faces decreased. To eliminate the response caused by non-specific binding, PBS buffer was injected into the capillaries to rinse out protein molecules that were physically absorbed on the GNPs surface. After the PBS buffer washing, a slowing trend and even a slight drop could be observed in the ROI responses of the sidewalls and fiber end-faces. A glycine solution (10 mM, pH = 3) was used to strip the surface-bound antibody (transferrin/IgG) solution samples to regenerate the sensing surface. 

**Figure 6:**
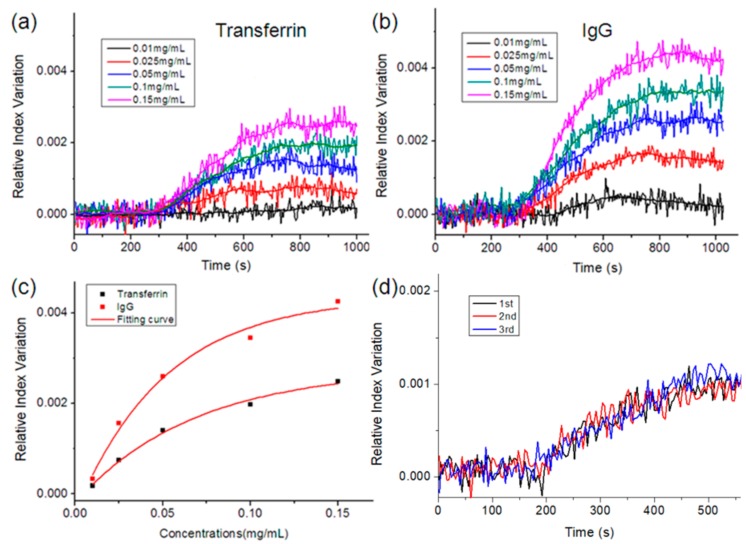
(**a**) Binding curve of the sensing device in transferrin detection; (**b**) Binding curve of the sensing device in IgG detection; (**c**) Relationship between the RI response and the concentrations; (**d**) Three measurement results of the binding process of IgG and protein A.

Figure 6a,b show the binding profile of transferrin-anti-transferrin/IgG-protein A at various concentrations. The results showed that as the sample concentration was increased, the change rate of relative intensity and signal responses became stronger. It could be explained that the RI near the GNP surface is determined by the adsorption quantity of protein molecules on the GNP surface. Therefore, as a larger number of antibody is captured by functionalized GNPs, higher RIs near the GNP surface can be expected. Since the RI near the GNP has an impact on the light scattering and absorption of the GNP, the relative intensities of the sidewall and fiber end-face changes with the samples and their concentrations. After fitting the data, we selected the mean of the relative intensity values from 800 s to 1000 s as the final response and plotted the corresponding RI–concentration curves. Figure 6c shows that the shapes of the fitting curves were similar to the logarithmic functions. We found that the final response increased with the rise of the sample concentrations, although the tendency slowed down. We also tested the reproducibility of the measurements. As shown in Figure 6d, the three binding profiles at the same concentration were roughly overlapped. In addition, the response values of the transferrin were less than that of IgG at the same concentration. This could be because the response was correlated to the density of the protein layer on the GNPs surface, and that the binding sites on the GNPs surface were limited. Since the molecular weight of transferrin was 77 kDa, half that of IgG (150 kDa), the response of IgG was much stronger than that of transferrin.

## 4. Conclusions

In summary, we demonstrated a simple strategy for the portable detection of human proteins using the LSPR effects of GNPs in capillaries, surface chemical modification techniques, and CMOS sensors. Since the surface of gold nanoparticles can be functionalized by various proteins, this novel sensing mechanism is suitable for the detection of many biochemical analytes. The combination of capillary LSPR sensors and CMOS image sensors made the sensing device easy to multiplex for the simultaneous detection of multiple analytes. In addition, based on the extinction of LSPR, the sensing signal was enhanced by detecting the scattered and transmitted light of the capillary device. We used this sensing device to detect transferrin and IgG by functionalizing the sensing surface, where the experimental results showed that the sensor response increased with the sample concentration. As a result, such a simple biosensing platform has the advantages of low cost, compactness, lightweight, and ease of use for the detection of biochemical analytes. This also has the potential to be used for protein detection outside the permanent laboratory.

## References

[1] Cetin A.E., Coskun A.F., Galarreta B.C., Huang M., Herman D., Ozcan A., Altug H. (2014). Handheld high-throughput plasmonic biosensor using computational on-chip imaging. Light Sci. Appl..

[2] Jha R., Sharma A.K. (2010). Design of a silicon-based plasmonic biosensor chip for human blood-group identification. Sens. Actuators B Chem..

[3] Wang L., Zhu Y., Xu L., Chen W., Kuang H., Liu L., Agarwal A., Xu C., Kotov N.A. (2010). Side-by-side and end-to-end gold nanorod assemblies for environmental toxin sensing. Angew. Chem. Int. Ed..

[4] Tseng S.Y., Li S.Y., Yi S.Y., Sun A.Y., Gao D.Y., Wan D. (2017). Food quality monitor: Paper-based plasmonic sensors prepared through reversal nanoimprinting for rapid detection of biogenic amine odorants. ACS Appl. Mater. Interfaces.

[5] Kailasa S.K., Koduru J.R., Desai M.L., Park T.J., Singhal R.K., Basu H. (2018). Recent progress on surface chemistry of plasmonic metal nanoparticles for colorimetric assay of drugs in pharmaceutical and biological samples. TrAC Trend. Anal. Chem..

[6] Hildebrandt A., Bragos R., Lacorte S., Marty J.L. (2008). Performance of a portable biosensor for the analysis of organophosphorus and carbamate insecticides in water and food. Sens. Actuators B Chem..

[7] Zhang D., Liu Q. (2016). Biosensors and bioelectronics on smartphone for portable biochemical detection. Biosens. Bioelectron..

[8] Roda A., Michelini E., Zangheri M., Di Fusco M., Calabria D., Simoni P. (2016). Smartphone-based biosensors: A critical review and perspectives. TrAC Trend. Anal. Chem..

[9] Iqbal M., Gleeson M.A., Spaugh B., Tybor F., Gunn W.G., Hochberg M., Baehr-Jones T., Bailey R.C., Gunn L.C. (2010). Label-free biosensor arrays based on silicon ring resonators and high-speed optical scanning instrumentation. IEEE J. Sel. Top. Quant..

[10] Brolo A.G. (2012). Plasmonics for future biosensors. Nat. Photonics.

[11] Pan S., Xu J., Shu Y., Wang F., Xia W., Ding Q., Xu T., Zhao C., Zhang M., Huang P. (2010). Double recognition of oligonucleotide and protein in the detection of DNA methylation with surface plasmon resonance biosensors. Biosens. Bioelectron..

[12] Zuo P., Li X., Dominguez D.C., Ye B.C. (2013). A PDMS/paper/glass hybrid microfluidic biochip integrated with aptamer-functionalized graphene oxide nano-biosensors for one-step multiplexed pathogen detection. Lab. Chip.

[13] Capitan-Vallvey L.F., Palma A.J. (2011). Recent developments in handheld and portable optosensing—A review. Anal. Chim. Acta.

[14] Srinivasan B., Tung S. (2015). Development and applications of portable biosensors. J. Lab. Autom..

[15] Wang X.D., Wolfbeis O.S. (2012). Fiber-optic chemical sensors and biosensors (2008–2012). Anal. Chem..

[16] Wang X.D., Wolfbeis O.S. (2015). Fiber-optic chemical sensors and biosensors (2013–2015). Anal. Chem..

[17] Shao Y., Xu S., Zheng X., Wang Y., Xu W. (2010). Optical fiber LSPR biosensor prepared by gold nanoparticle assembly on polyelectrolyte multilayer. Sensors.

[18] Gowri A., Sai V.V.R. (2016). Development of LSPR based U-bent plastic optical fiber sensors. Sens. Actuators B Chem..

[19] Rivero P.J., Urrutia A., Goicoechea J., Matias I.R., Arregui F.J. (2013). A lossy mode resonance optical sensor using silver nanoparticles-loaded films for monitoring human breathing. Sens. Actuators B Chem..

[20] Singh S., Mishra S.K., Gupta B.D. (2013). Sensitivity enhancement of a surface plasmon resonance based fibre optic refractive index sensor utilizing an additional layer of oxides. Sens. Actuators B Chem..

[21] Cennamo N., D’Agostino G., Pesavento M., Zeni L. (2014). High selectivity and sensitivity sensor based on MIP and SPR in tapered plastic optical fibers for the detection of L-nicotine. Sens. Actuators B Chem..

[22] He Y.J. (2015). Novel and high-performance LSPR biochemical fiber sensor. Sens. Actuators B Chem..

[23] Jin Y. (2012). Engineering plasmonic gold nanostructures and metamaterials for biosensing and nanomedicine. Adv. Mater..

[24] Petryayeva E., Krull U.J. (2011). Localized surface plasmon resonance: Nanostructures, bioassays and biosensing—A review. Anal. Chim. Acta.

[25] Kratz F., Elsadek B. (2012). Clinical impact of serum proteins on drug delivery. J. Control. Release.

[26] Chen G., Sequeira F., Tyan D.B. (2011). Novel C1q assay reveals a clinically relevant subset of human leukocyte antigen antibodies independent of immunoglobulin G strength on single antigen beads. Hum. Immunol..

[27] Buffone G.J., Lewis S.A. (1979). Manual immunochemical nephelometric assays for serum immunoglobulins IgG, IgA, and IgM. Clin. Chem..

[28] Sun L., Knierman M.D., Zhu G., Dovichi N.J. (2013). Fast top-down intact protein characterization with capillary zone electrophoresis–electrospray ionization tandem mass spectrometry. Anal. Chem..

[29] Zhou J., Gan N., Li T., Zhou H., Li X., Cao Y., Wang L., Sang W., Hu F. (2013). Ultratrace detection of C-reactive protein by a piezoelectric immunosensor based on Fe_3_O_4_@ SiO_2_ magnetic capture nanoprobes and HRP-antibody co-immobilized nano gold as signal tags. Sens. Actuators B Chem..

[30] Kuhla B., Albrecht D., Bruckmaier R., Viergutz T., Nürnberg G., Metges C.C. (2010). Proteome and radioimmunoassay analyses of pituitary hormones and proteins in response to feed restriction of dairy cows. Proteomics.

[31] Du D., Wang J., Lu D., Dohnalkova A., Lin Y. (2011). Multiplexed electrochemical immunoassay of phosphorylated proteins based on enzyme-functionalized gold nanorod labels and electric field-driven acceleration. Anal. Chem..

[32] Liu Y., Liu Q., Chen S., Cheng F., Wang H., Peng W. (2015). Surface plasmon resonance biosensor based on smart phone platforms. Sci. Rep..

[33] Liu Y., Chen S., Liu Q., Liu Z., Wei P. (2017). Simple method for self-referenced and lable-free biosensing by using a capillary sensing element. Opt. Express.

